# Eutrophication forcings on a peri-urban lake ecosystem: Context for integrated watershed to airshed management

**DOI:** 10.1371/journal.pone.0219241

**Published:** 2019-07-24

**Authors:** Annika E. Putt, Erland A. MacIsaac, Herb E. Herunter, Andrew B. Cooper, Daniel T. Selbie

**Affiliations:** 1 School of Resource and Environmental Management, Simon Fraser University, Burnaby, British Columbia, Canada; 2 Centre for Aquaculture and Environmental Research, Science Branch, Pacific Region, Fisheries and Oceans Canada, West Vancouver, British Columbia, Canada; 3 Cultus Lake Salmon Research Laboratory, Science Branch, Pacific Region, Fisheries and Oceans Canada, Cultus Lake, British Columbia, Canada; University of Nottingham, UNITED KINGDOM

## Abstract

Peri-urban lakes increasingly experience intensified anthropogenic impacts as watershed uses and developments increase. Cultus Lake is an oligo-mesotrophic, peri-urban lake near Vancouver, British Columbia, Canada that experiences significant seasonal tourism, anthropogenic nutrient loadings, and associated cultural eutrophication. Left unabated, these cumulative stresses threaten the critical habitat and persistence of two endemic species at risk (Coastrange Sculpin, Cultus population; Cultus Lake sockeye salmon) and diverse lake-derived ecosystem services. We constructed water and nutrient budgets for the Cultus Lake watershed to identify and quantify major sources and loadings of nitrogen (N) and phosphorus (P). A steady-state water quality model, calibrated against current loadings and limnological data, was used to reconstruct the historic lake trophic status and explore limnological changes in response to realistic development and mitigation scenarios. Significant local P loadings to Cultus Lake arise from septic leaching (19%) and migratory gull guano deposition (22%). Watershed runoff contributes the majority of total P (53%) and N (73%) loads to Cultus Lake, with substantial local N contributions arising from the agricultural Columbia Valley (41% of total N load). However, we estimate that up to 66% of N and 70% of P in watershed runoff is ultimately sourced via deposition from the nutrient-contaminated regional airshed, with direct atmospheric deposition on the lake surface contributing an additional 17% of N and 5% of P. Thus, atmospheric deposition is the largest single source of nutrient loading to Cultus Lake, cumulatively responsible for 63% and 42% of total N and P loadings, respectively. Modeled future loading scenarios suggest Cultus Lake could become mesotrophic within the next 25 years, highlighting a heightened need for near-term abatement of P loads. Although mitigating P loads from local watershed sources will slow the rate of eutrophication, management efforts targeting reductions in atmospheric-P within the regional airshed are necessary to halt or reverse lake eutrophication, and conserve both critical habitat for imperiled species at risk and lake-derived ecosystem services.

## Introduction

Peri-urban lakes near urban centres increasingly experience augmented watershed uses, modifications, and developments as suburban populations expand and commercial, recreational, and residential uses increase [1,2]. Ecosystem services provided by such lakes (e.g., fisheries, recreation, drinking water, irrigation) can be of significant cultural and socioeconomic value, with the conservation of water quality and ecosystem structure and functioning important for sustainable use [3,4]. Cultural eutrophication of freshwater ecosystems is a globally-pervasive, population-related problem, driven by excess external and internal nutrient forcings (i.e. phosphorus, nitrogen) that can threaten the water quality and ecology of affected lakes [5,6,7,8]. Cooperation among diverse stakeholders, multiple levels of government, and Indigenous communities is needed to develop and action long-term watershed-scale, and in some cases airshed-scale management plans that reflect all interests [9,10,11].

**Fig 1:**
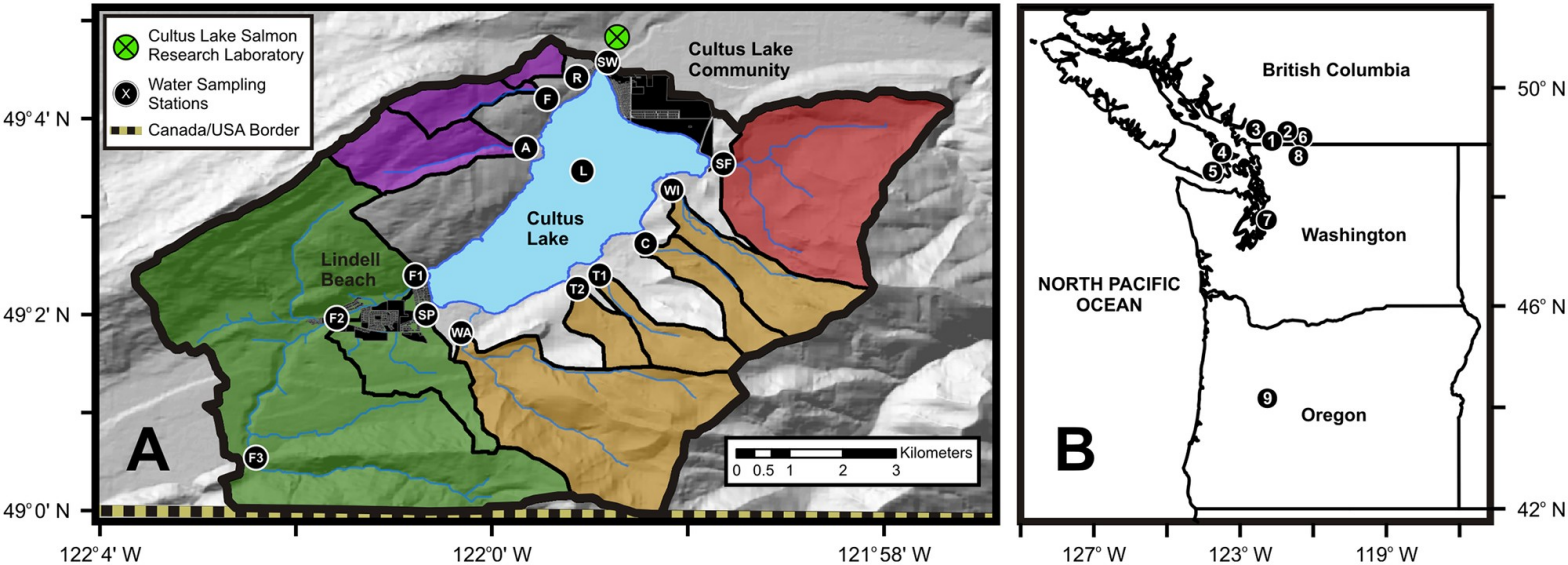
Map of the Cultus Lake, BC watershed (Panel A; Canadian portion shown) in relation to Pacific Northwestern North America (Panel B) and the locations of watersheds and lakes referenced in the text as data sources (Panel B: 1-Cultus Lake; 2-Elk Creek Watershed; 3-Malcolm Knapp Research Forest; 4-Saltspring Island; 5-Sooke Lake and Leech River; 6-Chilliwack Lake; 7-Lake Washington; 8-North Cascades National Park; 9-H.J. Andrews Experimental Forest). Locations of the Cultus Lake water sampling stations are shown in Panel A (R-Reservoir Creek; F-Fin Creek; A-Ascaphus Creek; F1-Frosst Creek Station 1; F2-Frosst Creek Station 2; F3-Frosst Creek Station 3; SP-Spring Creek, WA- Watt Creek; T1-Teapot Creek braid 1; T2-Teapot Creek braid 2; C-Clear Creek; WI-Windfall Creek; SF-Smith Falls Creek; SW-Sweltzer Creek (lake outlet); L-Fisheries and Oceans Canada limnological monitoring station), as is the location of the local atmospheric sampling station at the Fisheries and Oceans Canada Cultus Lake Salmon Research Laboratory. Cultus Lake tributary catchment boundaries are coloured to reflect sub-catchment agglomerations derived from clustering analysis as presented in the text, including Vedder Mountain (purple), Columbia Valley (green), International Ridge (orange), and Smith Falls Creek (red; Panel A). The outflow Sweltzer Creek subwatershed, largely encompassing the community of Cultus Lake is not coloured.

Cultus Lake, British Columbia, Canada is a peri-urban lake, proximate to several growing urban centers, experiencing eutrophication and associated water quality degradation [12]. The majority (82%) of its 69 km^2^ international watershed (Canada-USA) is located within the British Columbia Lower Mainland (Canada), ~80 km east of Vancouver, and ~10 km south of Chilliwack, adjacent to extensive agriculture, transportation corridors, and industries of the Fraser Valley (Fig 1). While the watershed only hosts ~1,000 permanent residents, it is a prized recreational and residential area, receiving 2 to 3 million visitors annually to its campgrounds, cottages and day-use areas [13,14]. Nearby Metro Vancouver and the Fraser Valley Regional District encompass ~2.9 million residents, a population expected to exceed 3.8 million people by 2040, with use and development of the watershed expected to increase proportionately [15].

Continued eutrophication is anticipated to negatively impact the ecology of Cultus Lake and its many lake-derived ecosystem services, affecting tourism and impairing local economies. Increased algal and macrophyte growth and associated decreases in water clarity and aesthetics would reduce recreational use [16,17,18], while habitat degradation and altered food webs may impair fish persistence and fisheries [16,19]. The lake serves as critical habitat for two fish species at risk: the threatened coastrange sculpin, Cultus population (*Cottus aleuticus*; [20,21]), which completes its life history within the lake, and the endangered Cultus Lake sockeye salmon (*Oncorhynchus nerka*; [22]), which uses the lake for adult spawning and juvenile rearing. Water quality degradation is known to contribute to the risk of extinction for both species, with eutrophication having the potential to disrupt physical, biological, and chemical aspects of their freshwater habitat [20,22,23,24]. For instance, eutrophication-related seasonal hypolimnetic oxygen depletion has at least doubled in Cultus Lake since the 1920’s -1930’s [12] and both species at risk rely significantly upon oxic profundal and benthic habitats to complete critical life functions.

Here we present a comprehensive study of watershed hydrology and watershed-to-airshed N and P fluxes to derive N and P budgets that facilitate nutrient abatement planning and management action for Cultus Lake. We developed a spatially-, seasonally-, and annually-resolved nutrient loading model, and undertook scenario-based lake modeling to 1) assess the current state of lake eutrophication and 2) examine the potential effects of development and watershed-scale mitigation strategies on lake water quality. Our study highlights a key environmental problem for freshwater ecosystems proximate to the British Columbia Lower Mainland and informs integrated watershed-to-airshed nutrient management for multi-use watersheds near expanding urban centers.

## Methods

### Study site

The Cultus Lake watershed (69 km^2^) is a transboundary catchment (82% Canada; 18% United States of America) located in the Cascade Mountains of southwestern British Columbia, Canada, and proximate to the Fraser Valley (Fig 1B). The single-basin (surface area 6.3 km^2^) warm monomictic lake has a mean depth of 31 m and a maximum depth of 44 m [12]. The basin is steep-sided with a limited littoral area (12% of the total area), more than 70% of which has been colonized by invasive Eurasian watermilfoil (*Myriophyllum spicatum L*.; [19,22]). The watershed is within the Coastal Western-Hemlock Biogeoclimatic Zone, characterised by a temperate maritime climate with mild winters and warm, dry summers [25]. Total annual precipitation averages 1,580 mm concentrated between the months of October and April [26]. Cultus Lake is currently oligo-mesotrophic, as indicated by the 2009–2012 mean growing season (April-November) euphotic zone chlorophyll *a* (chl-*a*; 2.22 μg/L ± 0.75 SD) and total phosphorus (TP; 8.00 μg/L ± 1.28 SD) concentrations, which yield Trophic State Index (TSI) values of 38 ± 3.26 SD for chlorophyll (TSI_Chl *a*_) and 34 ± 2.39 SD for phosphorus (TSI_TP_) [27].

Cultus Lake is bounded by the Vedder Mountain ridge to the west, International Ridge to the east, and the Columbia Valley to the south (Fig 1A). There are 11 major tributaries to Cultus Lake, the catchments of which drain 60% of the total watershed area, with the remainder drained by un-channelized runoff. We grouped the stream drainages into 5 subwatersheds based upon similar geomorphologies, land uses, and stream characteristics, using hierarchical agglomerative cluster analysis [28,29,30]. Details of the subwatershed geologies are given in S1 Text. The Vedder Mountain subwatershed encompasses 3 main tributaries (Ascaphus, Fin, and Reservoir creeks) and ungauged areas that drain the northwest lakeshore (Fig 1A), and is dominated by second-growth broadleaf species including bigleaf maple (*Acer macrophyllum*) and red alder (*Alnus rubra*) [31]. The International Ridge subwatershed, which includes Windfall, Clear, Teapot, and Watt creeks (Fig 1A) exhibits primarily coniferous forest, with 80% of the total area categorized as open or dense coniferous cover, dominated by western redcedar (*Thuja plicata*) and Douglas-fir (*Pseudotsuga menziesii*; [31]). Most of the International Ridge subwatershed lies within Cultus Lake Provincial Park, with campgrounds and day use areas along the shoreline, and upland recreational trails.

The largest subwatershed, the Columbia Valley, encompasses the southern extents of both constraining ridges and the valley bottom (Fig 1A), including coniferous forested uplands within the United States. The Columbia Valley is drained by 2 creeks, Frosst Creek, which is the largest inflowing tributary to Cultus Lake, and Spring Creek which emerges directly from the Columbia Valley aquifer near the lake. The slopes bounding the Columbia Valley are predominantly forested, while agriculture and other developments (20% of the total subwatershed area) occur along the central valley floor [31]. The community of Lindell Beach and numerous smaller developments bound the southern shore of Cultus Lake and Frosst Creek.

The Smith Falls Creek and Sweltzer Creek subwatersheds, drain the northern portion of the Cultus Lake watershed (Fig 1A). A portion of the Smith Falls Creek subwatershed lies within the densely forested Cultus Lake Provincial Park, while its north-eastern portion is sparsely covered and contains meadows and wetlands. The Sweltzer Creek subwatershed encompasses the community of Cultus Lake (population ~1,000) and the outlet of the lake, Sweltzer Creek, which flows northward along a shallow gradient into the Chilliwack River. Due to the northward sloping gradient, most drainage from this subwatershed (other than that directly proximate to the lake) likely flows subsurface to Sweltzer Creek and the Chilliwack River [32].

### Watershed water balance

We developed a hydrological budget for Cultus Lake to estimate hydrological and nutrient fluxes within the catchment. Tributary flows were monitored continuously over the two-year study period (May 2011 to May 2013), precipitation and evaporation data were obtained from nearby meteorological stations [30], and groundwater flows were estimated using a groundwater model developed for the watershed (Table 1) [32].

**Table 1:** Data sources used to estimate water balance and nitrogen (N) and phosphorus (P) nutrient budget inputs to Cultus Lake. Details and literature references are given in Methods and S1 Methods.

**Water balance data sources**	
Precipitation to the lake	Measured by precipitation gauge at the meteorological station near lake outlet (May 2011 to May 2013)
Lake evaporation	Used regional evaporation data from nearby meteorological station at Agassiz, British Columbia (May 2011 to May 2013)
Subwatershed tributary stream inflows and ungauged watershed runoff	Measured using hydrometric stations installed on tributary streams for major subwatersheds (May 2011 to May 2013). Ungauged subwatershed runoff interpolated from gauged subwatershed runoff.
Subwatershed groundwater inflows	Calculated as the difference between average annual areal runoff for the Cultus Lake watershed and areal runoff for each subwatershed
Lake outflow	Measured using hydrometric station at lake outlet
Groundwater outflow from the lake	Used results from a numerical groundwater model developed for Cultus Lake
**Nitrogen and Phosphorus nutrient data sources**	
Subwatershed runoff	Laboratory analyses of bi-weekly water samples collected from tributary streams and lake outlet (May 2011 to May 2013)
Subwatershed groundwater	Average tributary N and P concentrations applied to groundwater flows
Columbia Valley groundwater	Analyses of bi-weekly water samples from Spring Creek (direct aquifer outflow) (May 2011 to May 2013)
Wet atmospheric deposition	Weekly laboratory analyses of pooled precipitation samples collected at the meteorological station near the lake outlet (March 2012 to September 2013)
Dry atmospheric deposition	Estimated from the dry proportion of deposition measured at atmospheric monitoring sites in nearby Abbotsford and Chilliwack
Migratory gull guano	Guano nutrient concentrations from literature values and population size from visual surveys on the lake
Septic leachate	Estimated using regional population data, land use statistics, nutrient concentrations from provincial sewerage practice manuals, and soil retention coefficients from regional septic surveys and literature values
Sockeye salmon carcasses	Salmon escapement data and average wet weights from annual enumerations conducted by Fisheries and Oceans Canada. Percent N and P content from literature values for sockeye salmon.

We recorded stage-height continuously with hydrometric stations in 4 tributaries (Frosst Creek, Ascaphus Creek, Smith Falls Creek, and Watt Creek) and the lake outlet (Sweltzer Creek; Fig 1A), and instantaneous discharges were measured bi-weekly in all streams over the two study years. We calculated continuous discharges for monitored streams using log power stage-discharge rating curves [33], and estimated discharge from uninstrumented streams using linear regressions of instantaneous discharges with proximate continuously-monitored streams. Surface runoff from the ungauged portions of the subwatersheds was estimated using area-weighted average flows for all monitored streams in the subwatershed.

The total average areal runoff for the Cultus Lake watershed was determined as the sum of all annual outflows from the lake (Sweltzer Creek outflow, direct groundwater outflow, and evaporation) minus direct precipitation onto the lake surface, averaged over the area of the watershed. Direct groundwater outflow from the lake was estimated using results from a numerical groundwater flow model developed by Holding and Allen [32]. Groundwater runoff for each of the subwatersheds was then calculated as the difference between the estimated average areal runoff for the Cultus Lake watershed and the areal surface runoff estimated for each subwatershed (including streams and overland flow) from the hydrometric data.

### Watershed nutrient budget

We developed a nutrient budget for the Cultus Lake watershed that included loads from gauged and ungauged tributaries, ungauged overland flows, groundwater flows and interflows, atmospheric deposition, and conspicuous nutrient sources including migratory gull guano, septic leachate, atmospheric deposition, and sockeye salmon carcasses (see input sources summarized in Table 1 and detailed methods and parameters in S1 Methods). We estimated total nitrogen (TN) and total phosphorus (TP) loads in watershed runoff and in precipitation using bi-weekly water chemistry data collected at the outlet of the 11 main tributaries that flow into the lake. Frosst Creek was sampled in several locations to differentiate nutrient loading from upland forested areas and lowland agriculturally-influenced areas. Nutrient loading from septic leachate, background atmospheric deposition and areal runoff, and gull guano were estimated using a combination of literature values, regional monitoring data, and expert knowledge. Internal loading within the lake was accounted for within the empirical relationships used in the steady-state model used [34]. Nutrient losses via the outlet were sampled bi-weekly at Sweltzer Creek (Fig 1A), and in-lake attenuation was estimated as the difference between all nutrient influxes and effluxes.

### Steady-state lake nutrient modeling

We used the BATHTUB Simplified Techniques for Eutrophication Assessment & Prediction Model (version 6.1; [34,35]) to empirically estimate steady-state epilimnetic TP, TN, and chlorophyll *a* (chl-*a*) concentrations in Cultus Lake from external N and P loads, under a variety of past and future scenarios. The BATHTUB model has been extensively used and its performance and predictive abilities have been assessed by previous researchers [36]. We used annual water balance flow volumes and time-weighted TN and TP concentrations for nutrient loadings, and loading estimates for sources without volumetric inputs (i.e. septic leachate, gull guano, salmon carcasses) were generated using their respective total annual nutrient loads and a nominal inflow volume of 0.01 hm^3^/yr to satisfy model input requirements. Cultus Lake epilimnetic water quality data, collected in 2011 and 2012, were used to calibrate the nutrient sedimentation and chl-*a* model parameters, and specific conductivity was used as a conservative tracer to verify the Cultus Lake water balance [30].

Once calibrated against current conditions, the BATHTUB model was used to predict epilimnetic nutrient and chl-*a* concentrations under 4 scenarios: Pre-Disturbance—watershed conditions before significant anthropogenic influences (i.e., pre-disturbance by Euro-American settlement in the region); Current with Mitigation—a current watershed conditions scenario with reasonably-achievable local nutrient abatement strategies; Future—a development scenario projected 25 years into the future using expert-advised projections of population increases, land use changes, and gull population expansion; and Future with Mitigation—the 25-year future scenario with the abatement strategies from Current with Mitigation enacted. Detailed methods and rationales for the 4 scenarios are given in S2 Methods. Projected changes in each of the current nutrient sources are summarized for each scenario in Table 2.

**Table 2:** Summary of changes made to the nutrient loads from each nutrient source in the current conditions nutrient loading model for the pre-disturbance, current conditions with mitigation, and future conditions with or without mitigation nutrient loading scenarios modeled.

	Change in Cultus Lake Nutrient Source Relative to Current Conditions					
Scenario	Watershed Export (excluding agricultural and septic)	Columbia Valley Agricultural Runoff	Direct Atmospheric Deposition to Lake	Septic Leaching	Migratory Gull Guano	Sockeye Salmon Carcasses
**Pre-disturbance (before Euro-American settlement)**	N and P inputs reduced to background levels estimated using data from similar but pristine watersheds	Not present	N and P inputs reduced to background levels estimated using data from similar but pristine watersheds	Not present	Not present	Increased 425% based on average annual escapement from 1953–1962
**Current Conditions with Mitigation**	Unchanged	Reduced 50% based on adoption of modern agricultural techniques	Unchanged	Removed assuming 100% sewerage	Reduced 45% assuming effectiveness of non-lethal deterrents	Unchanged
**Future Development (25-year projection)**	Fraction of N and P attributed to anthropogenic sources increased by 30% based on 25-year population trend data	Increased 100% based on expected increase in agricultural activity in Columbia Valley	Increased by 30% based on 25-year population trend data	Increased septic leaching based on projected increases in residents and tourists	Increased 180% based on population trends from bird count data	Unchanged
**Future Development with Mitigation**	Unchanged from Future Development scenario	Same as Current Conditions scenario assuming expected increases in agriculture can be fully mitigated with modern techniques	Increased by 30% in line with population trend data for Cultus Lake and the Fraser Valley	Removed assuming 100% sewerage	Increased 180% based on population trends from bird count data then decreased 45% assuming effectiveness of non-lethal deterrents	Unchanged

## Results

### Water balance

Total inflow to Cultus Lake from watershed sources and direct precipitation was estimated at 110 hm^3^/yr (Table 3), with surface runoff to the lake accounting for 62% of total inflows and groundwater sources accounting for 30%. Groundwater constituted 45–51% of total runoff from the International Ridge, Vedder Mountain and Smith Falls Creek subwatersheds, high percentages due to the thin glacial tills and soil deposits on the steep bedrock slopes of these drainages. Extensive deposits of fractured rock, glacial tills, outwash sediments, and colluvial and alluvial sediments [32] yield rapid infiltration of surface runoff to the groundwater, resulting in relatively high groundwater inflows.

**Table 3:** Cultus Lake average annual water balance (May 2011-May 2013).

		Annual Average (m^3^/yr)	% Total
**INFLOW**			
***I***	**Subwatershed surface runoff**		
**Vedder Mountain**		6,401,307	5.8
**International Ridge**		14,850,751	13.5
**Smith Falls Creek**		5,264,991	4.8
**Columbia Valley**		41,891,643	38.0
***G***_***in***_	**Subwatershed groundwater inflow**		
**Vedder Mountain**		5,515,793	5.0
**International Ridge**		11,954,062	10.5
**Smith Falls Creek**		5,500,508	5.0
**Columbia Valley**		10,482,789	9.5
***P***	**Direct precipitation**	8,564,850	7.8
**OUTFLOW**			
***O***	**Sweltzer Creek outflow**	106,788,407	96.7
***G***_***out***_	**Groundwater outflow**	39,366	<0.1
***E***	**Evaporative loss**	3,629,755	3.3

The contribution of groundwater to total subwatershed runoff was lower in the Columbia Valley relative to the other subwatersheds. The Columbia Valley aquifer represents a large groundwater reservoir proximate to Cultus Lake, but 80% of total subwatershed runoff was accounted for in surface runoff via Frosst and Spring Creeks, rather than by subsurface groundwater discharge within the lake (Table 3). Spring Creek is a groundwater spring emerging near the lakeshore, and seasonal stream discharge patterns in Frosst Creek indicated groundwater inflows from the aquifer contribute substantially to baseflows during summer low flows. Most of the Columbia Valley groundwater appears to emerge from the aquifer as surface water at the valley bottom, thereby contributing to the surface runoff component of total subwatershed runoff.

### Nutrient budget

#### Atmospheric deposition of nutrients

Our measured wet atmospheric N and P deposition within the Cultus Lake watershed was elevated from spring to fall, with seasonal peaks that corresponded to the timing of early-summer and fall tillage and manure and fertilizer applications in the nearby agricultural Fraser Valley [37,38]. TN and TP concentrations in rainfall were low (<80 μg-N/L and <1 μg-P/L) from November through April but reached very high concentrations (1,200 μg-N/L and 60 μg-P/L) during the dry season of May through October. Peak TN concentrations in precipitation exceeded subwatershed stream concentrations, except for Ascaphus and Reservoir creeks on Vedder Mountain, but were below the peak concentrations measured in rainfall at sites proximate to agriculture sources in the Fraser Valley [38]. We measured peak rainfall TP concentrations that far exceeded values in any of the Cultus watershed streams and were more than double the peak levels measured in the nearby Elk Creek reference watershed in the eastern Fraser Valley (Fig 1B; [38]).

#### Subwatershed stream nutrient patterns

High atmospheric N and P deposition across the Cultus Lake watershed may in turn yield high TN and TP in runoff, particularly from subwatersheds nearest to and downwind of the major atmospheric nutrient sources in the Fraser Valley. Our estimates of mean annual flow-weighted (MAFW) TDN and TDP in runoff from each of the subwatersheds generally reflected local orographic effects and their proximity to atmospheric deposition sources in the Fraser Valley [39,40]. Ascaphus and Reservoir creeks, located in the most northern Vedder Mountain subwatershed of Cultus Lake, exhibited the highest mean annual flow-weighted (MAFW) TDN concentrations (averaging 557 and 690 μg-N/L TDN respectively), while the other tributaries ranged from 148 to 452 μg-N/L, suggesting atmospheric deposition of agriculturally-derived N from the Fraser Valley enriches runoff from the more northerly catchments. In contrast, the upland Frosst Creek water quality station (F3; Fig 1A), situated upstream of the influence of Columbia Valley agriculture and furthest from all sources of N emissions in the Fraser Valley, had the lowest average TDN levels of any of the Cultus Lake watershed streams at 79 μg-N/L.

Spatial patterns in TDP levels among streams were similar to those for TDN, with the highest concentrations occurring in subwatersheds closest to potential atmospheric sources in the Fraser Valley. Creeks draining the three Vedder Mountain subwatersheds and the Smith Falls Creek subwatershed had the highest MAFW TDP concentrations of all the tributaries at 9.3 to 13.0 μg-P/L, with the International Ridge subwatershed streams intermediate at 7.3 to 9.6 μg-P/L. As with TDN, the lowest TDP concentrations were recorded in the upper Frosst Creek in the upper Columbia Valley subwatershed (F3, Fig 1A) at 6.9 μg-P/L.

Hierarchical Frosst Creek sampling confirmed that agricultural N contamination of Columbia Valley groundwater is a major source of N loading to Cultus Lake [41]. Peak TDN concentrations were consistently lower upstream of agricultural influences (site F3 in Fig 1A; maximum 183 μg-N/L), relative to concentrations downstream of agricultural influences (site F2 in Fig 1A; maximum TDN >800 μg-N/L). The downstream station exhibited the highest TDN levels of any stream during late-summer low flows, when groundwater inputs accounted for nearly 100% of Frosst Creek baseflows. Columbia Valley well-water sampling provided further evidence that agricultural N contamination of Columbia Valley groundwater (mean 458 μg-N/L, maximum 1,890 μg-N/L) is the source of elevated TDN for lower Frosst Creek and the groundwater-sourced Spring Creek (maximum TDN >300 μg-N/L), the two major sources of runoff from the valley to Cultus Lake.

Unlike TDN, seasonal TDP variations in the Columbia Valley subwatershed were similar to those of the other subwatersheds. TDP concentrations at the Frosst Creek stations upstream and downstream of agricultural influences were very similar, with TDP seasonally ranging from 3 to 18 μg-P/L in the upstream, forested station (F3, Fig 1A) and 4 to 14 μg-P/L in the valley bottom (F2, Fig 1A) indicating that, unlike N, Columbia Valley groundwater is not significantly enriched with P by local agriculture.

#### Subwatershed areal nutrient export rates

Congruent with the elevated levels of TDN and TDP recorded in their tributary streams, export rates of TP from the Vedder Mountain and Smith Falls Creek subwatersheds were 0.31–0.32 kg P/ha/yr, higher than rates for the International Ridge and Columbia Valley subwatersheds (Table 4). The average areal TP export rate for the entire Cultus watershed was 0.26 kg P/ha/yr. Given a background export rate of 0.065 kg P/ha/yr from the nearby Chilliwack Lake watershed (i.e., a relatively pristine watershed; see S2 Methods), atmospheric deposition may be contributing ~70% of the TP exported in runoff to the lake. Given its geologic similarity to the Chilliwack Lake watershed, it is unlikely there are large unknown phosphate sources in the Cultus Lake watershed such as deposits of phosphate-bearing rocks contributing substantial mineral-weathered P. This implies that atmospheric TP deposition largely originating from regionally-enriched atmospheric sources is the major TP source in watershed runoff. Accounting for background deposition, the contribution of atmospheric TP from the subwatersheds combined with direct deposition on the lake accounts for 42% of the total P load to Cultus Lake.

**Table 4:** Average annual areal export rates of total nitrogen (TN) and total phosphorus (TP) in surface and groundwater runoff for the Cultus Lake watershed and each sub-watershed and annual atmospheric deposition rates to the lake.

Source	Average Annual Areal Export (kg/ha/yr)	
	TP	TN
**Average Watershed Export**	0.26	6.01
**Vedder Mountain**	0.31	8.33
**International Ridge**	0.19	4.53
**Smith Falls Creek**	0.32	4.51
**Columbia Valley**	0.23	6.54
**Atmospheric Deposition to Lake Surface**	0.20	13.73

Export rates of TN in runoff from each of the subwatersheds reflected both differences in land use and cover among subwatersheds, in addition to their proximity to atmospheric deposition sources of the Fraser Valley (Table 4). The highest N export occurred from the Vedder Mountain subwatershed (8.33 kg-N/ha/yr), which is closest to N emission sources in the Fraser Valley. The Smith Falls Creek subwatershed had the lowest TN export rate (4.51 kg-N/ha/yr), despite its proximity to the Fraser Valley, which could reflect significant N retention by the extensive upslope wetlands unique to this catchment. In comparison, nitrogen exports are typically much lower (≤ 2 kg-N/ha/yr) in similar but pristine watersheds in Pacific Northwestern North America [42,43,44]. Combined with direct deposition to the lake surface and accounting for background TN deposition, atmospheric TN is responsible for 63% of the total N load to Cultus Lake.

#### Total nutrient loads to Cultus Lake

Overall TN loading to the lake was estimated to be 51 tonnes-N/yr, with 73% of the TN load coming from surface and groundwater runoff from the watershed (Table 5). The largest single source of TN loading was from the Columbia Valley subwatershed, which delivered 21 tonnes-N/yr or 41% of the overall TN load, largely via surface runoff enriched with agricultural N by groundwater admixture. Nitrogen from wet and dry atmospheric deposition directly onto the lake surface contributed 8.7 tonnes-N/yr or 17% of the total N load, a high proportion given that the lake surface area is about 10% of the total watershed area. Near-shore septics were also an important source of N loading to the lake and contributed 9% to the overall TN load. Gull guano and salmon carcasses were minor sources of TN to the lake (<2% of the total load). Approximately 57% of the TN load was retained within the lake, presumably attenuated by elevated autotrophic production [12] and exports to the lake sediments.

**Table 5:** Annual total nitrogen and phosphorus loads into Cultus Lake. Totals for individual subwatersheds are broken into surface water and groundwater loads, and septic leaching is separated into the two subwatersheds with the most septic systems. Lake outflow exports via Sweltzer Creek are also shown.

	Average Annual Load (kg/yr)	
Source	TP	TN
**Vedder Mountain Total**	**221 (8.1%)**	**5,978 (11.8%)**
Surface	118	3,217
Groundwater^a^	103	2,761
**International Ridge Total**	**305 (11.1%)**	**7,310 (14.4%)**
Surface	164	3,750
Groundwater^a^	141	3,560
**Smith Falls Creek Total**	**209 (7.6%)**	**2,923 (5.8%)**
Surface	101	1,449
Groundwater^a^	107	1,474
**Columbia Valley Total**	**724 (26.4%)**	**20,643 (40.7%)**
Surface	582	16,418
Groundwater	142	4,225
**Atmospheric Deposition to Lake Surface**	**124 (4.5%)**	**8,673 (17.1%)**
Measured wet deposition	83	3,573
Estimated dry deposition	42	5,100
**Migratory Gulls**^b^	**614 (22.4%)**	**476 (0.9%)**
**Septic Leaching**	**523 (19.1%)**	**4,557 (9.0%)**
Columbia Valley Septics	170	1,485
International Ridge Septics	67	586
Cultus North Septics	285	2,487
**Sockeye Carcasses**	**23 (0.8%)**	**191 (0.4%)**
**TOTAL LOAD**	**2,744**	**50,751**
Sweltzer Creek Export	-1,258	-21,952
% Retention	54.2%	56.7%

^a^Includes shallow soil interflow

^b^Total Kjeldahl Nitrogen (TKN; excludes NO_3_^-^-N which is expected to be low in guano)

Overall TP loading to Cultus Lake was estimated to be 2,744 kg-P/yr with 53% loaded via surface and groundwater runoff from the subwatersheds (Table 5). Septic leaching and migratory gull guano contributed 523 and 614 kg-P/yr respectively, a combined 42% of the total annual P load. Gull guano is a substantial P input to Cultus Lake in the fall and winter seasons when gulls raft on the lake overnight, while leaching of phosphate from septic systems occurs along the littoral zone primarily during the peak summer visitation period, when the lake is thermally stratified. Direct atmospheric deposition on the lake surface was also a significant source of P loading to surface waters, contributing 124 kg-P/yr or about 5% of the total P load. However, considering that more than half of the P delivered by runoff from the subwatersheds may originate from atmospheric deposition on the landscape, atmospheric deposition is a large and underappreciated source of P loading to Cultus Lake. Overall, 54% of the total TP load was retained within the lake.

### Steady-state water quality modeling

**Fig 2:**
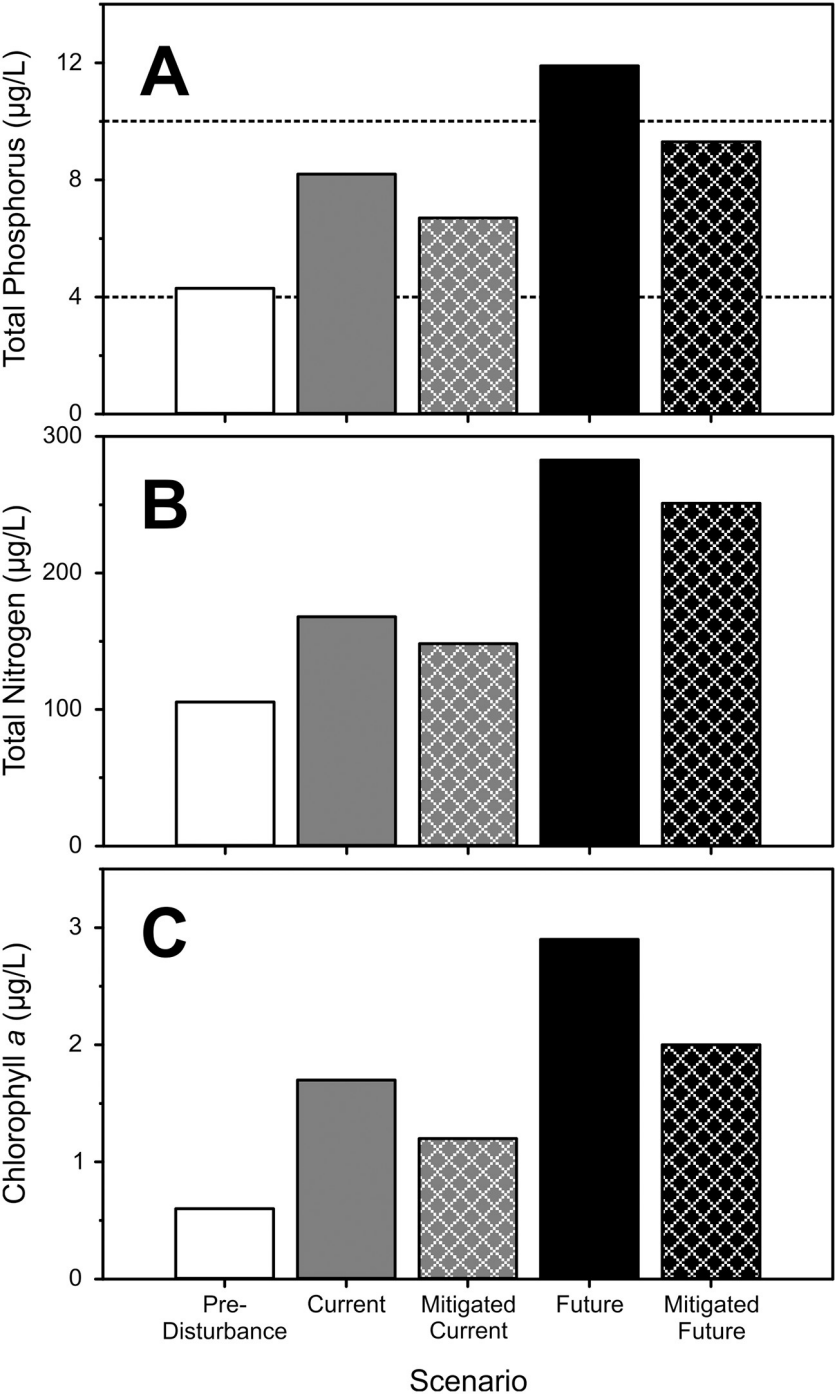
BATHTUB model results for current steady state water quality conditions in Cultus Lake (grey bars), the estimate of water quality conditions prior to anthropogenic disturbance of the watershed and airshed (white bars), predicted water quality with current nutrient sources mitigated (grey bars with hash marks), and water quality predictions for two future development scenarios without nutrient mitigation (black bars) and with mitigation (black bars with hash marks). TP (Panel A), TN (Panel B), and chl-a (Panel C) are steady-state model estimates of epilimnetic growing season averages. Dotted reference lines in Panel A indicate the Canadian Council of Ministers of Environment thresholds [45] for TP-inferred aquatic system trophic status (lower line—ultra-oligotrophy-oligotrophy threshold (4 μg/L TP); upper line—oligotrophy-mesotrophy threshold (10 μg/L TP)).

The BATHTUB model [34,35] was calibrated against current lake conditions and used to hindcast the trophic status of Cultus Lake prior to substantial anthropogenic influences on the watershed and airshed, and to predict lake responses to watershed nutrient management scenarios now and under future development. With minimal calibration, the model accurately estimated current epilimnetic TN, TP, and chl-*a* concentrations observed within Cultus Lake (Table 6). The trophic status of Cultus Lake was oligotrophic [45] under both pre-disturbance and current conditions but has significantly eutrophied from its natural state prior to Euro-American settlement (increased TP +91%, TN +59%, and chl-*a* +183%; Fig 2). Epilimnetic TP concentrations have doubled from a pre-disturbance seasonal mean value of 4.3 μg-P/L, to current concentrations of 8.2 μg-P/L, primarily due to the effects of migratory gull guano, septic leaching, and anthropogenically-influenced atmospheric P deposition (largely delivered in runoff). Epilimnetic TN has increased from 106 to 168 μg-N/L, largely resulting from agricultural loadings from the Columbia Valley and anthropogenically-influenced atmospheric N deposition.

**Table 6:** Calibration results for the BATHTUB model with observed and predicted epilimnetic concentrations of TP, TN, and chl-*a* for Cultus Lake (growing season averages), calibration factors, and descriptions of the model equations used.

Parameter	Observed^a^	Predicted^a^	Calibration Factor	Model Equation
**TP (μg/L)**	8.25	8.25	0.87	Second order available phosphorus
**TN (μg/L)**	167.98	167.98	0.80	Second order available nitrogen
**Chl-*a* (μg/L)**	1.68	1.68	0.95	Exponential as a function of phosphorus

^a^Epilimnetic growing season averages; mean of 2011 and 2012

If future development and increases in nutrient loadings proceed as projected over the next 25 years, without targeted nutrient mitigation, the model predicts 66%, 68% and 106% increases in epilimnetic TP, TN and chl-*a* concentrations, respectively (Fig 2). Increased nutrient loadings from septic systems, migratory gulls, and atmospheric deposition will further eutrophy the lake, increasing mean growing season TP to 13.6 μg-P/L and shifting the lake to a mesotrophic state [45].

Reasonably-achievable mitigation targets applied to nutrient loads from the three watershed nutrient sources that were feasible in the short-term (reduced septic -100%, agriculture -50% and gull -45%) elicited moderate water quality improvements for Cultus Lake. Epilimnetic TP, TN and chl-*a* concentrations were predicted to decrease by 20%, 12%, and 29%, respectively from current conditions (Fig 2). Reductions in epilimnetic TP primarily arose from elimination of septic system leaching, one of the largest local sources of P loadings to the lake, while reduced agricultural loadings from the Columbia Valley largely accounted for the TN reductions. Modeled water quality improvements were limited because two of the largest nutrient sources to the lake, direct atmospheric deposition and subwatershed runoff (heavily influenced by atmospheric deposition), can only be mitigated through broader regional nutrient management, beyond the scope of local watershed abatement strategies.

When the nutrient mitigation measures used in the mitigation scenario were applied to the future development scenario, the model still predicted further degradation of water quality relative to current conditions (Fig 2). However, declines in water quality were less pronounced than predicted for the unmitigated future development scenario. Future epilimnetic TP, TN and chl-*a* concentrations were predicted to increase by 27%, 49%, and 41% respectively, compared to the 66%, 68% and 106% increases predicted without mitigation. As such, localized nutrient abatement measures can moderate the expected impacts on the water quality of Cultus Lake arising from future development.

## Discussion

### Principal nutrient forcings on Cultus Lake, BC

Cultus Lake, BC is a highly valued peri-urban aquatic ecosystem hosting multiple species at risk that is experiencing degrading water quality from nutrient enrichment [12]. Our nutrient budget and modeling highlight that eutrophication of Cultus Lake over the past century [12] has been forced by a spatially and temporally complex combination of diffuse and point nutrient sources.

We identified diffuse loading of nutrients via atmospheric deposition to the lake and watershed from the regionally-contaminated airshed [39] as the primary trophic status driver for Cultus Lake. Although watershed export contributed the largest portion of the total N (73%) and P (53%) load to the lake, our nutrient budget and modeling suggests airshed forcings on Cultus Lake nutrient status are much more profound, with atmospheric nutrient deposition contributing the majority of the N (66%) and P (70%) in watershed export. Direct deposition to the lake surface also contributed substantial N (17%), and to a lesser extent P (5%) to the total load to the lake.

Contamination of groundwater and surface runoff by agricultural sources in the Columbia Valley was also a large source of N loading to Cultus Lake, with the subwatershed contributing 41% of the N load in total watershed runoff. Nitrate levels in Frosst Creek were seasonally variable and elevated when the proportion of agriculturally-contaminated groundwater was high during late summer low flows, and hierarchical sampling indicated substantial elevation of N downstream of the agricultural valley. Nitrates have been shown to freely leach into groundwater through deep glacial outwash sediments similar to those of the Columbia Valley [46,47], suggesting elevated lability for local agricultural N leaching into groundwater exports to the lake. Export from the Columbia Valley subwatershed was also a large portion of the total P loading to Cultus Lake (26%), but groundwater P exports were not commensurate with those of N. While elevated P exports have been well documented for surface runoff from agricultural catchments [48,49,50,51], the lack of P enrichment in Columbia Valley groundwater indicates agriculturally-sourced P is retained in the glacial sediments during the deep (i.e. 30–50 m) percolation to the aquifer [41]. Unlike N, phosphates are readily sorbed and retained by iron and aluminum oxides and clay and calcium minerals characteristic of glacial sediments [46,51], suggesting Columbia Valley substrates are at present unsaturated and retaining agricultural P. The capacity of the Columbia Valley to retain further agricultural P loadings is unknown and there may be a risk of enhanced P loading to Cultus Lake from continued or expanded agricultural activities or shifting of focal crops (i.e. increased P fertilizer applications).

Migratory gull guano (22% of total TP load) was a substantial seasonal source of P loading to Cultus Lake, but a minor source of N. Guano P loading to the lake surface was 5-fold higher than direct atmospheric deposition of P. Septic leaching was also an important local source of both N (9%) and P (19%) loads to Cultus Lake annually. Septic leaching is particularly prevalent in the watershed due to the advanced age of many systems, and the close proximity of large communal septic fields to the lake. Salmon carcasses were not a significant source of N and P at present.

### Cultus Lake nutrient status past, present and future

Our steady-state lake nutrient and watershed modeling identifies the primary forcings underpinning the current eutrophication trajectory of Cultus Lake and predicts current and future lake outcomes with and without local-scale nutrient abatement. Consistent with inferences from centennial-scale comparative limnology [12] and local observations of degrading water quality [52], our model indicated Cultus Lake has substantially eutrophied from its natural oligotrophic state. Our findings are supported by limnological research in 2001–2003, indicating enhanced lake productivity and pronounced seasonal hypolimnetic oxygen depletion (-50%) relative to the 1920’s-1930’s [12,53], common symptoms of ongoing lake eutrophication [6]. While abatement of local nutrient loadings to Cultus Lake are essential to recover water quality, our models predict only modest improvements to the current lake condition, owing to the dominant atmospheric control on nutrient loadings from the watershed.

Lake nutrient stresses arising from an expanding regional population, and associated use of the watershed, are projected to increase significantly over the next 25 years. Our models predict unmitigated future development impacting the watershed and regional airshed will increase nutrient loadings and lake nutrient concentrations commensurately from current levels. Cultus Lake is predicted to shift into a mesotrophic state (11.9 μg-P/L, [45]), with considerably higher algal biomass. Similarly, without targeted abatement of regional atmospheric sources, the application of reasonably-achievable local watershed nutrient mitigation measures only moderate water quality degradation from current levels, resulting in an oligo-mesotrophic lake state (9.3 μg-P/L).

### Consequences of further eutrophication within Cultus Lake, BC

Future algal biomass increases in Cultus Lake would reduce water clarity, likely shifting seasonal algal production higher in the water column, commensurately impacting lake ecology and aesthetics [12]. Enhanced P-loading may induce proximate N-limitation in surface waters [54], which can stimulate the formation of heterocystous cyanobacteria blooms, as have been observed sporadically in Cultus Lake (i.e. *Anabaena* spp.). Cyanobacterial blooming would further amplify lake organic matter (OM) loads and negatively impact tourism and associated economies [18,55]. Increased OM sedimentation would exacerbate existing hypolimnetic oxygen deficits, reducing the quality and availability of deep-water habitats, and potentially altering aquatic food webs for species at risk (i.e. Coastrange Sculpin and Sockeye Salmon) [20,24,56]. Without nutrient abatement, anoxia at the sediment-water interface, which has been observed in areas of Cultus Lake in the late-summer and fall, is likely to increase, facilitating internal loading of nutrients and other redox-sensitive contaminants from lake sediments, which could rapidly accelerate and reinforce lake eutrophication and seasonal hypolimnetic oxygen depletion [57].

Future eutrophication of Cultus Lake is likely to be exacerbated by climate change [8,9,58]. Warmer air temperatures and reduced summer precipitation [59] will likely yield stronger and protracted lake stratification [7], enhancing the effects of septic leaching and possibly gull guano loading on water quality and algal production. Lake surface warming and enhanced hypolimnetic oxygen depletion could create a “temperature-oxygen squeeze” whereby increasingly hypoxic hypolimnetic waters and thermally sub-lethal to lethal epilimnetic waters encroach upon one another, degrading and reducing available habitat for cold-water fish species [60]. Cultus Lake Sockeye Salmon already face persistence threats from overexploitation [61] and increasing river temperatures during migration [62]. Further eutrophication and hypolimnetic oxygen depletion would increase the risk of extinction for this species and the threatened Coastrange Sculpin (Cultus Population), which require profundal and benthic habitats to complete critical life functions [62,63].

### Prescriptions for lake management

Lake eutrophication is a reversible phenomenon, and mitigation of watershed nutrient sources has elicited near-complete water quality recovery in regional systems where local sources comprise the bulk of loadings (e.g., Lake Washington; [64]). Local mitigation is essential to resolving cumulative, excess nutrient loadings to Cultus Lake while broader airshed mitigation strategies can be developed and enacted. In particular, a focus on abating phosphorus inputs will elicit the greatest reductions in lake productivity and associated habitat degradation, improving water quality [12]. Addressing the primary, local P-loads (i.e. septic leaching, migratory gull guano deposition, local agricultural runoff) will retard the rate of lake eutrophication and must be a near-term management focus for Cultus Lake to preserve societal valuations, species at risk, and ecosystem services.

Septic leachate is a significant global driver of eutrophication for lakes with intensive shoreline development [5,65,66], with abatement of septic loadings significantly improving water quality [67,68]. Reducing septic leachate via sewerage removal, or at least enhanced treatment [69], will be essential to mitigating eutrophication within Cultus Lake. Similarly, avian guano loading can disrupt nutrient cycling in temperate lakes, resulting in eutrophication [70,71], and our models indicate significant reductions in P loading and algal production with guano reductions. Reducing avian occupation will be challenging, as glaucous-winged gulls are protected under the Migratory Bird Convention Act [72], attracted to rich feeding areas in the Fraser Valley, and common noise-related deterrence and lethal control methods are not publicly favourable [72,73]. A variety of non-lethal tactics (i.e. pyrotechnic scaring devices, blank ammunition, and gull distress calls) have successfully reduced gull numbers at landfills and agricultural areas [73,74,75], and should be considered for daytime feeding sites, with realizable improvements in water quality expected.

Conservation agricultural practices (i.e. soil-specific fertilizer dosing, modified fertilization application timing, low tillage practices, minimizing annual bare soil exposure, perennial cropping systems) have reduced nutrient loading to lakes [76,77,78]. Water quality improvements from agricultural abatements applied within the Columbia Valley are predicted to reduce N-loading, but may not affect P-loading. Reducing local agricultural runoff is a key eutrophication mitigation strategy, but must be accomplished with concurrent P-reduction strategies to avoid unintended induction of surface N-limitation and heterocystous cyanobacteria blooms. Such local-scale nutrient mitigation measures can be readily applied to the Cultus Lake watershed, without the need for substantial cross-jurisdictional cooperation, and would be highly-beneficial for the near-term preservation of water quality, lake-derived ecosystem services, and species at risk.

### Atmospheric nutrient deposition within regional watersheds

Atmospheric nutrient deposition from the regionally contaminated airshed [39] is a first-order nutrient forcing on Cultus Lake and a potentially overlooked ecological driver of regional peri-urban aquatic ecosystems. Elevated atmospheric nutrient contributions likely reflect prevailing westerly atmospheric flows, transporting pollutants from upwind sources (i.e. Metro Vancouver, Fraser Valley, Whatcom County, USA) up the Fraser Valley into adjoining mountain valley watersheds [40,79]. The coincidence of peak N and P concentrations in Cultus Lake precipitation with seasonal soil tillage, and fertilizer and manure applications in the Fraser Valley is congruent with deposition within the Fraser Valley agricultural belt [37,38]. This highlights the key role of agriculture in contaminating the regional airshed and recipient watersheds, with influences extending well beyond the agricultural zone.

Elevated atmospheric deposition of nutrients recorded throughout the British Columbia Lower Mainland indicate our observations are likely of broader concern, as urban and agricultural emissions are likely impacting highly valuable aquatic ecosystems throughout the region [40]. Enhanced N deposition has been documented in the nearby city of Abbotsford (8.6 kg-N/ha/yr), and in several undeveloped watersheds bordering the Fraser Valley, including the Elk Creek drinking water reference watershed (25.7 kg-N/ha/yr) [34,38], and the East Creek (~6 kg-N/ha/yr) and Loon Lake watersheds within the undeveloped University of British Columbia Malcolm Knapp Research Forest [80,81]. Soil N-saturation from decades of anthropogenic N deposition in the East Creek watershed [81], suggests nutrient emissions are altering regional biogeochemical cycling in terrestrial and aquatic ecosystems.

Anthropogenic N deposition is a pervasive low-level global nutrient pollutant in aquatic ecosystems, impacting even pristine watersheds far from emissions sources [80,82,83]. Where focussed, however, it can be a major nutrient loading to watersheds, particularly downwind of urban and agricultural regions [48,84]. N deposition exceeds 20 kg-N/ha/yr across most of the northeastern United States [85]. While western North America hosts fewer intensive urban and agricultural sources, deposition within urban airsheds can also surpass 20 kg-N/ha/yr [86,87]. The cumulative effects of such loadings may increase N saturation in terrestrial and aquatic ecosystems, altering nutrient stoichiometry in lakes, and changing biodiversity [80,82,84,88,89].

Anthropogenic N deposition can alter the nature of nutrient limitation in aquatic ecosystems (i.e. P vs. N) and may be leading to widespread chronic P-limitation in lakes [65], making them highly responsive to P-loadings and other forcings (i.e. climate change). While information on regional atmospheric P-deposition is scarce, our modeling indicates it has a prolific influence on direct and watershed-mediated P-exports to Cultus Lake, which is P-limited or P- and N-co-limited throughout most of the growing season [12]. Given the relative importance of P in forcing eutrophication [8,90] and the magnitude and extent of regional N deposition [39], atmospheric P deposition is likely a primary driver of regional aquatic ecosystem changes, and a key target for abatement not currently considered in regional flux models [91]. Moreover, by virtue of their geographical position, watershed geologies and extents, and prevailing atmospheric flows, Lower Mainland aquatic ecosystems may be disproportionately responsive to atmospheric P loadings from the contaminated Fraser Valley airshed.

The magnitude and extent of regional atmospheric nutrient deposition will continue to degrade Cultus Lake and other regional aquatic ecosystems, should primary emission forcings on the shared airshed not be addressed. Given its lack of a stable gaseous phase, atmospheric transport and deposition of P largely occurs via entrainment of aerosols [90] which may be a helpful constraint both in identifying and minimizing emissions, and reducing regional P deposition. While all P sources to the regional airshed need to be identified and quantified in order to focus abatement priorities, agriculture is unarguably a dominant forcing of Fraser Valley P flows [91]. We hypothesize that optimization (i.e. soil-specific dosing) of the significant amounts of P applied to the regional landscape via agriculture [91], coupled with targeted interruptions to seasonal aerosol entrainment (i.e. modified low- or no-tillage practices, minimization of bare soil land exposure, reduced liquefied manure spraying, reductions in poultry barn exhaust bio-aerosols) could substantially reduce atmospheric P loading to the regional airshed and regional aquatic ecosystems. An integrated landscape-to-airshed nutrient management approach inclusive of stakeholders, governments, and Indigenous communities, and their individual and shared values, will be essential to reduce airshed P deposition, and halt or reverse eutrophication trends at Cultus Lake and across the region. Failure to do so could result in significant impacts on cultural and socioeconomic valuations, lake-derived ecosystem services, and species at risk.

## Supporting information

S1 Text Cultus Lake watershed geology. (DOCX)

S1 Methods Estimation of nutrient loads. (DOCX)

S2 Methods Development of scenarios. (DOCX)

S1 Datasets Project datasets. (ZIP)
