# Supplementary material for: Eutrophication forcings on a peri-urban lake ecosystem: Context for integrated watershed to airshed management
Source: PLoS One. 2019 Jul 24;14(7):e0219241. doi: 10.1371/journal.pone.0219241 (PMC6655610; doi:10.1371/journal.pone.0219241)
Supplement: S1 Text — (DOCX) [file pone.0219241.s001.docx]

**S1 Text: Cultus Lake watershed geology**

Cultus Lake is bounded by the Vedder Mountain ridge to the west, International Ridge to the east, and the Columbia Valley to the south and the two constraining ridges and the Columbia Valley have very different geologic and hydrologic characteristics. The bedrock of Vedder Mountain is primarily metasedimentary sandstone, conglomerate, and shale while International Ridge is primarily underlain by slatey-argillite [1]. Both are overlain by shallow glacial gravel tills and rapidly-drained sandy organic soils [2,3]. The surficial geology in the Columbia Valley is characterized by glaciofluvial sand and gravel outwash sediments (part of the Sumas Drift deposit) that are greater than 120 m deep [1,2]. The unconfined Columbia Valley aquifer is formed by these deep outwash sediments and extends from a topographic groundwater divide near the international border to the Cultus Lake shoreline [1]. The Columbia Valley features a raised terrace of outwash deposits that abruptly drops ~ 700 m southwest of Cultus Lake. The terrace is bisected by a 50-m deep gorge cut through the sediments by Frosst Creek as it flows from International Ridge on the south-east side, across the Columbia Valley to the lake.

**References**

1. Zubel M, Eng P. Groundwater Conditions of the Columbia Valley Aquifer Cultus Lake, British Columbia. Report prepared for the Ministry of Environments, Lands, and Parks Water Management. 2000 Jan.; 98p. Available from: http://a100.gov.bc.ca/appsdata/acat/documents/r6477/1287_1143682706883_8b7182b483ed458aad9da29ff7cef555.pdf
2. Holding S, Allen D. Cultus Lake watershed numerical groundwater flow model. Simon Fraser University, Burnaby (BC). 2012. Available from: <https://www.psf.ca/sites/default/files/FSWP_11_34_FR_Attachment_Cultus_Lake_Groundwater_Model_Report.pdf>
3. MapPlace. Surficial Geology Map Index; British Columbia Geological Survey MapPlace website, BC Ministry of Energy, Mines and Petroleum Resources; 2014 [cited 18 Mar 2014]. Available from <http://webmap.em.gov.bc.ca/mapplace/minpot/OF_13map.cfm>
