# Supplementary material for: Eutrophication forcings on a peri-urban lake ecosystem: Context for integrated watershed to airshed management
Source: PLoS One. 2019 Jul 24;14(7):e0219241. doi: 10.1371/journal.pone.0219241 (PMC6655610; doi:10.1371/journal.pone.0219241)
Supplement: S1 Methods — (DOCX) [file pone.0219241.s002.docx]

**S1 Methods: Estimation of nutrient loads**

**Stream and atmospheric chemistry and load estimation**

Total nitrogen (TN) and phosphorus (TP) loading from tributary inflows and direct precipitation were estimated using weighted sample mean averaging, where instantaneous loads (i.e., concentration multiplied by discharge) were scaled by average discharges over the relevant time period (see equations in Table 1) [1]. We sampled stream water chemistry at the outflow of each of the 11 main tributaries into Cultus Lake and the Sweltzer Creek outflow on a biweekly basis from May 2011 to May 2013 (Fig 1A). In Frosst Creek, 3 stations (F1, F2, F3; Fig 1A) were sampled along a transect over the stream reach to determine water quality parameters as the creek flowed from the forested mountain ridge through agricultural lands and various developments to the lake.

Replicate water samples were syringe-filtered in the field through sample-washed glass-fibre filters (Pall Acrodisc, 1.0 μm) for analysis of dissolved organic matter (DOM), dissolved organic nitrogen (DON), nitrate-nitrite (NO3-N), ammonium (NH4+-N), dissolved inorganic phosphorus (DIP), and total dissolved phosphorus (TDP). Particulate phosphorus (PP) and particulate nitrogen (PN) samples were filtered onto ashed borosilicate microfiber filters (equivalent to a Whatman GFF) and frozen. Total phosphorus (TP) was calculated as the sum of TDP and PP and total nitrogen (TN) was the sum of DON, PN, NO3-N and NH4+-N. Water and particulate chemical analyses were performed at Fisheries and Oceans Canada’s Cultus Lake Salmon Research Laboratory [2,3].

Mean subwatershed tributary nutrient concentrations were used as average values for ungauged and groundwater inflows in each of the subwatersheds, and Spring Creek was used to estimate groundwater chemistry for the Columbia Valley (Fig 1A). We also sampled groundwater directly in 3 Columbia Valley wells (British Columbia well tag numbers 18149, 37208, and 92975) to validate that Spring Creek was representative of groundwater throughout the valley. A two end-member mixing model of conductivity and DOM for Frosst Creek separated seasonal groundwater and surface water source contributions for this primary lake inflow [4].

We sampled precipitation chemistry at the meteorological station (Fig. 1A) to estimate wet N and P deposition, using 25-cm diameter funnels in light-excluded vessels (year 2 only). Pooled weekly precipitation samples were analyzed for DIP, TDP, NO3-N, and NH4+-N, following the analytical methods used for the stream samples. The sum of NO3-N, and NH4+-N was used as the wet deposition TN estimate. TDP was considered equivalent to wet deposition TP. Commensurate dry deposition of N and P was estimated from the dry proportion of deposition measured at Environment and Climate Change Canada (ECCC) atmospheric monitoring sites in nearby Abbotsford and Chilliwack [5,6], which were assumed to be similar to Cultus Lake. The sum of wet and dry deposition was used as the total N and P atmospheric deposition to the lake surface. DON was not measured for the wet TN atmospheric fraction. Thus, the total N atmospheric deposition estimate is likely biased low but is comparable to the ECCC deposition measurements.

**Biotic vector nutrient load estimation**

We estimated nutrient loading to Cultus Lake arising from migratory gull (primarily glaucous-winged gulls; Larus glaucencens) guano deposition during nighttime roosting on the lake (Table 1). Resident observations and data from the National Audubon Society Christmas Bird Count (CBC) in Chilliwack indicate gull populations in the area were historically minimal but have increased since at least the late-1970’s [7,8]. Gulls diurnally migrate to and from the Cultus Lake watershed from late-fall to early-spring, feeding at agricultural fields, waterbodies, and waste disposal sites in the Fraser and Chilliwack River valleys, returning to raft on the lake overnight, where they excrete nutrients. To estimate guano loading, we integrated annual population estimates of 9,500-12,500 gulls from visual surveys and existing literature values of nutrient production rates for herring gulls (L. argentatus), a close relative of glaucous-winged gulls [9,10,11,12]. Literature values of daily gull nutrient production are highly variable (0.12 to 0.66 g/gull/d for total Kjeldahl nitrogen (TKN), and 0.24 to 1.82 g/gull/d for TP), so we calculated a median load, based on the literature range.

Sockeye salmon (Oncorhynchus nerka) carcasses represent a natural nutrient loading to Cultus Lake, whereby primarily marine-derived nutrients are imported upon migration from the ocean to the lake (i.e. anadromy) and are released upon death (i.e. semelparity) following spawning in the fall. We estimated nutrient loads from salmon carcasses using historic escapement data from Fisheries and Oceans Canada (DFO), nutrient contents of 3.04% for N and 0.36% for P by weight [13], and the average weight (minus gonad weight) of adult Cultus Lake sockeye salmon carcasses (1.55 kg, 2010 data; Patterson D, DFO, unpublished data; Table 1).

**Septic leachate nutrient loading estimation**

Leaching of nutrients into Cultus Lake occurs from diffuse private septic systems, centralized on-site wastewater treatment systems arising from numerous small communities and resort developments in the Columbia Valley subwatershed, large provincial campgrounds located along the shoreline of the International Ridge subwatershed, and the residences and Sunnyside Campground at the north end of the lake. Although groundwater modeling [14] predicts northward subsurface flows away from the lake, water quality testing of the Main and Sunnyside Campground beaches (Sweltzer Creek subwatershed) identified summer Escherichia coli concentrations indicative of septic leaching (historic data provided by Fraser Health). Thus, the Sweltzer Creek subwatershed septic systems within 600 m of the shoreline were included in the septic leaching estimate. Septic systems in the watershed use buried leaching fields, with high nutrient leaching potentials due to the shallow depth of the water table relative to the depth of the fields, proximity to watercourses that flow into Cultus Lake, low filtration capacities of the coarse-textured gravelly sub-soils, the potential for nutrient saturation in aging septic systems and soils, and the high precipitation and leaching rates in the region [15,16,17].

We estimated nutrient loads from septic systems in the Columbia Valley, International Ridge, and the northern subwatersheds using sewage flow rates obtained from the British Columbia Sewerage Standards Practice Manual for residential (175 L/cap/d), campground (90 L/cap/d), day-use areas (20 L/cap/d; [18]), and sewage outflow nutrient concentrations measured in the Okanagan region of BC (32 mg/L for TN and 7.8 mg/L for TP; [19]; Table 1). Residential inflows were calculated as the flow rate for a 3-person dwelling reduced by a peaking factor of 50%. We used recently-derived residential population estimates, residence totals, and seasonal occupancy rates for the Columbia Valley [17] and Cultus Lake North residences [20]. Camping and day use statistics for Cultus Lake Provincial Park [21], Sunnyside Campground and Main Beach were used to inform septic loads in these high-use areas (Fraser Valley Regional District, unpublished). We used conservative substrate retention coefficients of 15% for TN and 60% for TP, based upon available soil composition data for the watershed [22] and substrate retention coefficients from the Spokane Valley-Rathdrum Prairie Aquifer in northeastern Washington, which has similar soil characteristics to the Cultus Lake watershed [23].

**Table 1. A summary of the equations and input parameters used to calculate nutrient loads for the steady-state Bathtub model.**

| Nutrient Input | Equation | Input Values |
| --- | --- | --- |
| Gauged and ungauged flow, groundwater flow and shallow interflow and wet deposition | ${load}_{t{rib}_{i}}=\frac{\sum_{n=1}^{N} C_{n}Q_{n}}{\sum_{n=1}^{N} Q_{n}}\bar{Q}$   \| ${load}_{{trib}_{i}}$ \| Average annual nutrient load for tributary *i* (kg/yr) \| \| --- \| --- \| \| $C_{n}$ \| Nutrient concentration in trib *i* at time *n* \| \| $Q_{n}$ \| Discharge in trib *i* at time *n* \| \| $\bar{Q}$ \| Average of all discharge measurements in trib *i* \| \| *N* \| Total number of sampling events \| | 1. Gauged tributaries: Sampled bi-weekly in tributaries May 2011 to May 2013 2. Ungauged flows: Interpolated as the average of subwatershed tributaries sampled in 1 3. Shallow groundwater flow: $C_{n}$ interpolated from average subwatershed tributaries sampled in 1; $Q_{n}$ was the difference between the total annual areal runoff for Cultus Lake and the subwatershed total annual areal runoff 4. Columbia Valley groundwater: $C_{n}$ sampled bi-weekly in Spring Creek (direct aquifer outflow) May 2011 to May 2013; $Q_{n}$ calculated as for 3 5. Wet deposition: Monitored continuously at lake outlet March 2012 to September 2013 |
| Dry deposition | ${load}_{dry dep.}=\frac{{load}_{wet}*{prop}_{dry}}{1-{prop}_{dry}}$   \| ${load}_{dry dep.}$ \| Annual dry deposition load (kg/yr) \| \| --- \| --- \| \| ${load}_{wet}$ \| Nutrient load calculated for wet precipitation (kg/yr) \| \| ${prop}_{dry}$ \| Proportion of total atmospheric load attributed to dry deposition \| | \| ${prop}_{dry}$ (TN) \| 62% \| \| --- \| --- \| \| ${prop}_{dry}$ (TP) \| 50% \| |
| Migratory gull guano | ${load}_{gull}=H_{G}*r$  $H_{G}=D*H*G$   \| ${load}_{gull}$ \| Annual load from migratory gulls (kg/yr) \| \| --- \| --- \| \| $H_{G}$ \| Total gull hours (hrs/yr) \| \| $r$ \| Production rate of migratory gulls (kg/hr) \| \| $D$ \| Number of days of occupation \| \| $H$ \| Hours of occupation per day \| \| $G$ \| Cultus Lake migratory gull population size (gulls/night) \| | \| $r$(TN) \| 0.12-0.66 kg/yr \| \| --- \| --- \| \| $r$ (TP) \| 0.24-1.82 kg/yr \| \| $D$ \| 181 (September 15 to March 15) \| \| $H$ \| 12 (1830 to 0630) \| \| $G$ \| 11,000 (visual surveys) \| |
| Septic leachate | ${load}_{{septic}_{i}}=N*Q_{pc}*C*(1-r)$   \| ${load}_{septic}$ \| Annual septic load from source *i* (kg/yr) \| \| --- \| --- \| \| $r$ \| Nutrient soil nutrient retention coefficient \| \| $N$ \| Annual person days (campers and day users) \| \| $Q_{pc}$ \| Per capita daily sewage flow rate (L/cap/day) \| \| $C$ \| Nutrient concentration in sewage output \| | \| $r$ (TN) \| 15% \| \| --- \| --- \| \| $r$ (TP) \| 60% \| \| $N$ \| Local population and camping data \| \| $Q_{pc}$ residential \| 175 L/cap/day \| \| $Q_{pc}$ campground \| 90 L/cap/day \| \| $Q_{pc}$ day-use \| 20 L/cap/day \| \| $C$ (TN) \| 32 mg/L \| \| $C$ (TP) \| 7.8 mg/L \| |
| Sockeye salmon carcasses | ${load}_{sockeye}=w*E*p$   \| ${load}_{sockeye}$ \| Annual nutrient load from sockeye salmon carcasses (kg/yr) \| \| --- \| --- \| \| $w$ \| Weight of one sockeye salmon spawner (kg) \| \| $E$ \| Estimated sockeye salmon escapement (mean escapement from 2011-2012) \| \| $p$ \| Percent nutrient content per wet weight \| | \| $w$ \| 1.55 kg \| \| --- \| --- \| \| *p* (TN) \| 3.04% \| \| *p* (TP) \| 0.36% \| \| *E* \| 3,187 fish \| |

**References**

1. Walling DE, Webb W. The reliability of suspended sediment load data [River Creedy, England]. In Erosion and Sediment Transport Measurement (Proceedings of the Florence Symposium); IAHS Publ. 1981;133: 177–194.
2. Stainton MP, Capel MJ, Armstrong FA. The chemical analysis of fresh water, 2nd ed. Can Fish Mar Serv Misc Spec Publ. 1977;25: 166 p.
3. Stephens K, Brandstaetter R. A laboratory manual: collected methods for the analysis of water. Can Tech Rep Fish Aquat Sci. 1159. 1983. Available from: <http://publications.gc.ca/collections/collection_2013/mpo-dfo/Fs97-6-1159-eng.pdf>
4. Putt, AE. Spatiotemporal nutrient loading to Cultus Lake: Context for eutrophication and implications for integrated watershed-lake management. Report No. 591. School of Resource and Environmental Management, Faculty of Environment, Simon Fraser University. 2014. Available from <http://rem-main.rem.sfu.ca/theses/PuttAnnika_2014_MRM591.pdf>
5. Belzer W, Evans C, Poon A. Atmospheric nitrogen concentrations in the Lower Fraser Valley. Aquatic and Atmospheric Sciences Division. Environmental Conservation Branch, Environment Canada.1997:201-401.
6. Vingarzan R, Belzer W, Thompson B. Nutrient levels in the atmosphere of the Elk Creek Watershed, Chilliwack, BC (1999-2000). Environment Canada technical report. 2002. Aquatic and atmospheric sciences division, Vancouver, Canada. Available from: http://www.pyr.ec.gc.ca/GeorgiaBasin/reports/NAECWC/GB-02–038_E.pdf
7. National Audubon Society. The Christmas bird count historical results [Online]. 2010 [cited 13 Apr 2014]. Available from: <http://www.christmasbirdcount.org>
8. Robinson M. Cultus Lake Aquatic Stewardship Strategy (CLASS) 5-year report. Fraser Basin Council. 2012. Available from: <http://cultusstewards.ca/wp-content/themes/Vizio-1.7.1/images/media/report-FSWP-2012.pdf>
9. Gould DJ, Fletcher MR. Gull droppings and their effects on water quality. Water Res. 1978;12: 665–672.
10. Portnoy JW. Gull contributions of phosphorus and nitrogen to a Cape Cod kettle pond. Hydrobiologia. 1990;202: 61–69. doi:10.1007/BF00027092
11. Marion L, Clergeau P, Brient L, Bertru G. The importance of avian-contributed nitrogen (N) and phosphorus (P) to Lake Grand-Lieu, France. Hydrobiologia. 1994;279: 133–147.
12. Hahn S, Bauer S, Klaassen M. Estimating the contribution of carnivorous waterbirds to nutrient loading in freshwater habitats. Freshwater Biol. 2007;52(12): 2421–2433. doi:10.1111/j.1365-2427.2007.01838.x
13. Larkin BGA, Slaney PA. Implications of trends in marine-derived nutrient influx to south coastal British Columbia salmonid production. Fisheries. 1995;22(11): 16-24.
14. Holding S, Allen D. Cultus Lake watershed numerical groundwater flow model. Simon Fraser University, Burnaby (BC). 2012. Available from: <https://www.psf.ca/sites/default/files/FSWP_11_34_FR_Attachment_Cultus_Lake_Groundwater_Model_Report.pdf>
15. Zubel M, Eng P. Groundwater Conditions of the Columbia Valley Aquifer Cultus Lake, British Columbia. Report prepared for the Ministry of Environments, Lands, and Parks Water Management. 2000 Jan.; 98p. Available from: http://a100.gov.bc.ca/appsdata/acat/documents/r6477/1287_1143682706883_8b7182b483ed458aad9da29ff7cef555.pdf
16. McCray JE, Kirkland SL, Siegrist RL, Thyne GD. Model parameters for simulating fate and transport of on-site wastewater nutrients. Ground Water. 2005;43(4): 628–39. doi:10.1111/j.1745-6584.2005.0077.x
17. Urban Systems Ltd. Cultus Lake South Sewerage Planning Study. Prepared for the Fraser Valley Regional District; USL File No. 0999.0042.01. Chilliwack (BC). 2012. Available from: <https://www.yumpu.com/en/document/view/41666790/cultus-lake-south-sewerage-planning-study-fraser-valley-/3>
18. British Columbia Ministry of Health. Sewerage System Standard Practice Manual. Victoria (BC). 2006. Available from: <http://www2.gov.bc.ca/gov/content/environment/waste-management/sewage/onsite-sewage-systems/sewerage-system-standard-practice-manual>
19. Kennedy GF, Oldham WK. Task report on the estimate of nutrient contribution to receiving waters from agricultural sources via groundwater transport and estimate of total nutrients reaching receiving waters in groundwater. Task 139. Prepared for the Okanagan Study Committee in Connection with the Canada-British Columbia Okanagan Basic Agreement. Penticton (BC). 1972.
20. ISL Engineering and Land Services. Cultus Lake sanitary sewer infrastructure assessment. Report submitted to the Fraser Valley Regional District, September 2012. Report No. 30825. 2012.
21. British Columbia Parks. 2011/12 Statistics Report. Victoria (BC). 2012.
22. Comar VK, Sprout PN, Kelley CC. Soil Survey of Chilliwack Map Area. Kelowna (BC): Agriculture and Agri-Food Canada. Preliminary Report #4, Lower Fraser Valley Soil Survey, B.C; B.C. Department of Agriculture, Kelowna, B.C., July 1962.
23. HDR Consulting. Spokane County onsite sewage disposal system phosphorus loading estimate technical memorandum. Prepared for: Spokane County. Spokane (WA). 2006. Available from: <https://www.spokanecounty.org/DocumentCenter/Home/View/2001>
