# Supplementary material for: Eutrophication forcings on a peri-urban lake ecosystem: Context for integrated watershed to airshed management
Source: PLoS One. 2019 Jul 24;14(7):e0219241. doi: 10.1371/journal.pone.0219241 (PMC6655610; doi:10.1371/journal.pone.0219241)
Supplement: S2 Methods — (DOCX) [file pone.0219241.s003.docx]

**S2 Methods: Development of scenarios**

To model the pre-disturbance and future development scenarios, background non-anthropogenic atmospheric deposition rates for TN and TP were required. While no true modern analog for pre-anthropogenic atmospheric deposition likely exists due to diffuse global atmospheric distribution of environmental contaminants [1,2], we used N and P deposition rates measured or modelled within pristine regions of Pacific Northwestern North America (PNWNA) as surrogates. Background atmospheric N loading was estimated using the annual average TN deposition rate (wet and dry) of 2.0 kg-N/ha/yr (1996-2007; range 1.4-2.7 kg-N/ha/yr) from the Clean Air Status and Trends Network (CASTNET; <http://epa.gov/castnet/javaweb/index.html>) monitoring site NCS415, located approximately 70 km southeast of Cultus Lake in the North Cascades National Park of Washington State (Fig 1B) [3]. This deposition rate corresponds with other modeled and measured estimates for areas of the PNWNA not heavily influenced by atmospheric contamination, including southwest British Columbia (<2.1 kg-N/ha/yr; [4]), and the western Cascade Range of coastal Oregon (H.J. Andrews Research Forest; 2.0-2.1 kg-N/ha/yr; [5]; Fig 1B)), and contrasts markedly with recent atmospheric N-deposition rates of 25.7 kg-N/ha/yr measured in the nearby agricultural region and transportation corridor of the Fraser Valley [6]. More limited, proximal information is available for P deposition rates within pristine PNWNA watersheds. As such, we used the H.J. Andrews Research Forest (Fig 1B) multi-station 10-year average (2000-2009) TP wet deposition rate (0.02 kg-P/ha/yr; [7]) and applied the 1:1 wet to dry deposition relationship recorded in the nearby Elk Creek watershed (Fig 1B) of the Fraser Valley [6] to estimate a background wet and dry TP atmospheric deposition rate of 0.04 kg-P/ha/yr for the Cultus Lake watershed. Our estimation is commensurate with downscaled global P deposition model outputs for natural deposition for the PNWNA [8], and is markedly lower than the 0.15 kg-P/ha/yr atmospheric P-deposition rate measured for the nearby agriculturally-impacted Fraser Valley [6].

Estimates of background areal N and P nutrient export rates (i.e. kg/ha/yr) for the Cultus Lake watershed and subwatersheds were also required to partition out the anthropogenic contributions to measured areal export rates for use in the pre-development and future development model scenarios. The average areal TN export rate of 2 kg-N/ha/yr recorded for 2 mature forested watersheds on Vancouver Island (Leech River and Sooke Lake watersheds; Fig 1B), which exhibit similar climates and forest covers to the Cultus Lake watershed, were adopted in our study [9]. N-deposition modeling and lichen biomonitoring confirm these Vancouver Island watersheds are relatively unaffected by regional anthropogenically-influenced N deposition [4], and the conservative estimation of 2 kg-N/ha/yr is generally corroborated by export rates measured in undisturbed forested watersheds in the H.J. Andrews Research Forest (Fig 1B; <1.5 kg-N/ha/yr; [5,10]).

Background areal P export rates to lakes can vary widely among watersheds owing to the geologically-specific nature of P-bearing mineral deposits and weathering, with literature estimates generally lacking for pristine PNWNA watersheds. As such, the surrogate background areal P export rate used in our study was derived through application of the BATHTUB model to limnological data from nearby Chilliwack Lake (Fig 1B), located within a similar biogeoclimatic setting 41 km east of Cultus Lake that is not heavily influenced by anthropogenic sources [4]. Annual P concentration and hydrologic data were used to calculate a steady state areal P export rate for the Chilliwack Lake watershed. This locally-estimated P export rate used for the Cultus Lake watershed of 0.065 kg-P/ha/yr was similar to P export rates measured from two relatively undisturbed watersheds (Roberts Lake (0.08 kg P/ha/yr); Blackburn Lake (0.10 kg P/ha/yr)) on Saltspring Island, BC (Fig 1B) [11].

**Pre-disturbance scenario**

To model the Cultus Lake trophic state prior to regional Euro-American settlement and associated anthropogenic inputs from the watershed (i.e., septic and agricultural inputs, export changes due to deforestation) and the regional airshed (i.e., vehicle and industrial emissions, agricultural volatilization, dust emissions), the estimated background watershed areal export rates of N and P to Cultus Lake were used in the BATHTUB model. Gull guano inputs were excluded as long-term residents report use of lake by migratory gulls is a relatively recent phenomenon [12] in agreement with available bird count data collected since the late 1970’s [13]. While other aquatic birds would have likely been resident at Cultus Lake prior to Euro-American settlement, without the attraction to anthropogenic food sources in the Fraser Valley they likely primarily fed and recycled nutrients within the watershed. To estimate pre-disturbance N and P loading from historically more abundant salmon carcasses, the average annual escapement for the earliest 10 years on record (1953 to 1962) was used.

**Future development scenario**

We forecasted N and P loads to Cultus Lake 25 years into the future as a scenario reflecting expected increases in watershed use, development and regional anthropogenic nutrient loads. Nutrient loads from atmospheric deposition to the lake and watershed, new and aging septic systems, and intensified agriculture are expected to increase as regional populations are grow by >40% over the next 25 years [14]. In this scenario, we scaled both direct atmospheric N and P deposition and the fraction of subwatershed TN and TP runoff loading attributable to anthropogenic sources by +30% to account for increased population density mitigated by expected advancements in pollution capture technologies. Although potentially influential, future changes in seasonal precipitation and hydrologic patterns projected with climate change were not explicitly incorporated into the future scenario water balance.

Christmas Bird Count (CBC) data for the Chilliwack area [13] and the North American Breeding Bird Survey for the coast region [14] both indicate long-term increasing trends in gull populations. As carrying capacity is unlikely to constrain future populations over the projected time range, we fit a linear time trend model (R^2^ = 0.36, p < 0.001, n = 32) to logarithm-transformed CBC count data from the Chilliwack area, which predicted a current gull population size of 9,030 gulls/yr, similar to the bird count estimates from this study (9,500 to 12,500 gulls/yr [15]), and a 180% population increase to 30,800 gulls/yr over the next 25 years, to which future TN and TP loads were scaled. Annual adult Sockeye Salmon carcass loads were estimated at current scenario levels, given significant uncertainty in stock rebuilding.

Future septic loads for the Cultus lake watershed were projected assuming continued use of discharge-to-ground technologies over the next 25 years. Columbia Valley septic loads were increased in proportion to a projected residential unit increase of 804 dwellings [16]. Similarly, in the North Cultus Lake area septic loads were increased in relation to an additional 341 dwellings, commensurate with a predicted full-time resident increase of ~700 persons [17]. While Cultus Lake Provincial Park visitation has been relatively stable in recent years [18], day visitation will likely increase over the next 25 years as a result of projected population growth in the nearby Fraser Valley (i.e., a 47% increase from 2001 levels by 2030; [19]). As such, we increased total day use septic loading from these campgrounds by 45%. We did not increase septic loading from camping use at the Cultus Lake Parks Board Sunnyside Campground, as there were no known planned campground expansions, but Main Beach loadings were increased by 1.5% annually compounded over 25 years to match the growth rate predictions (+45% over 25 years) used for the Cultus Lake Provincial Park public use areas.

Agricultural loading from the Columbia Valley is likely to increase as land use projections suggest an increase in total cultivated area and a shift towards more intensive forms of agriculture including berries, vegetables, and intensive forage (Sutherland K., BC Ministry of Agriculture, August 2018, pers. comm). Current land use statistics indicate that approximately 45% of the total cropped area is intensive crops with nutrient leaching potential, including ~15% as berries and ~30% as intensive forage (BC Ministry of Agriculture 2016). Although it is difficult to project cropping trends 25 years in the future, expert opinion conservatively indicates berry production could double to 30% of the total cropped area, while natural cover and non-intensive pasture may decrease by 40% (from 40% to 25% of total cropped area) and replaced with intensive forage (Sutherland K., BC Ministry of Agriculture, August 2018, pers. Comm; BC Ministry of Agriculture 2016). We determined Columbia Valley agriculture-specific nutrient loading by calculating areal export rates for the portions of the watershed located upstream and downstream of agricultural influences, using nutrient and hydrologic data for the two Frosst Creek stations that bound the Frosst Creek flow pathway through the agriculturally-influenced portion of the subwatershed (F2, F3; Fig 1A). The difference between these rates was assumed to be the agricultural contribution (10% and 73% of total TP and TN loads respectively) and was doubled to reflect the aforementioned increases in intensive agricultural activity.

**Current conditions with mitigation scenario**

We explored the combined effects of applying a suite of three reasonably-achievable local watershed nutrient mitigation strategies on the current trophic conditions of Cultus Lake: diversion of all septics to sewerage, non-lethal migratory gull deterrence, and improved agricultural nutrient management in the Columbia Valley. Only nutrient mitigation measures that could be implemented within a local Cultus Lake watershed management initiative were considered as mitigation of atmospheric nutrient deposition from anthropogenic sources outside of the watershed would require major provincial and federal government efforts to affect reductions in nutrient emissions from agricultural, automotive and industrial sources in the lower mainland of British Columbia. Any atmospheric nutrient deposition that might originate from agricultural or other anthropogenic sources within the Cultus Lake watershed was ignored as the contributions from small local sources are likely dwarfed by large scale regional emissions [4].

Septic nutrient loads were removed entirely assuming septics in the watershed could be diverted to a sewer system and exported from the watershed for treatment, the most stringent option currently under review by the Fraser Valley Regional District, which also includes aggressive nutrient abatement with discharge to ground [16].

As gulls are protected under the Migratory Birds Convention Act (MBCA), and public acceptance of lethal culling is generally low and uncommon [20], non-lethal methods of deterring gulls were explored, assuming they could be discouraged from using the lake. Falcons, non-lethal shot, and gull distress calls have resulted in 40% to 50% long-term reductions in gull numbers at landfill sites [21,22,23], and assuming similar results could be achieved, gull guano nutrient loading was reduced by 45% in the current mitigation scenario.

Implementation of modern methods of fertilizer and manure management practices in the Columbia Valley could feasibly reduce current nutrient leaching into surface and groundwater runoff from berry crops by 70% to 80% and from forage and hobby farms by up to 50% (Sutherland K, BC Ministry of Agriculture, March 2014, pers. comm.). The current portion of the areal nutrient export in runoff attributed to agriculture in the Columbia Valley was thus reduced by 50%, assuming modern agricultural nutrient management practices could be applied throughout the Columbia Valley. While delays are likely in water quality improvements after septic and agricultural loading reductions due to soil retention processes, for simplicity all mitigation measures were assumed to have immediate effects on lake water quality.

**Future development with mitigation scenario**

A final scenario was examined that combined the increased nutrient loads to Cultus Lake expected over the next 25 years in the future development scenario with the reasonably-achievable nutrient mitigation measures outlined in the current conditions with mitigation scenario (i.e. septic leachate, bird guano, and agriculture loading reductions). As with the current conditions with mitigation scenario, only nutrient mitigation measures that could be implemented within a local Cultus Lake watershed management initiative were considered. Septic leaching was eliminated assuming all septic systems could be diverted to sewerage out of the Cultus Lake watershed. Predicted gull nutrient loads in the future development scenario were reduced by 45%, assuming effective non-lethal deterrence. Although agricultural loadings were expected to double, implementation of modern fertilizer and manure management techniques could reduce total agricultural loadings by ~50%, and thus future agricultural loadings under mitigation were held at current levels. Atmospheric deposition to the lake was increased by 30%, as was the portion of subwatershed runoff attributed to atmospheric deposition, as it was assumed technological improvements may partially offset increased atmospheric loads from a population increase of 40% in the Fraser Valley. Sockeye salmon carcass loads were left unchanged from the future development scenario.

**References**

1. Elser JJ. A world awash in nitrogen. Science. 2011;334: 1504-1505.
2. Holtgrieve GW, Schindler DE, Hobbs WO, Leavitt PR, Ward EJ, Bunting L, et al. A coherent signature of anthropogenic nitrogen deposition to remote watersheds of the Northern Hemisphere. Science. 2011;334: 1545-1548.
3. Fenn ME, Ross CS, Schilling SL, Baccus WD, Larrabee MA, Lofgren RA. Atmospheric deposition of nitrogen and sulfur and preferential canopy consumption of nitrate in forests of the Pacific Northwest, USA. Forest Ecol and Manag. 2013;302: 240–253. doi:10.1016/j.foreco.2013.03.042
4. Raymond BA, Bassingthwaighte T, Shaw DP. Measuring nitrogen and sulphur deposition in the Georgia Basin, British Columbia, using lichens and moss. J Limnol. 2010;69(Suppl. 1): 22–32. doi:10.3274/JL10-69-S1-04
5. Martin CW, Harr DR. Precipitation and streamwater chemistry from undisturbed watersheds in the cascade mountains of Oregon. Water Air Soil Poll. 1988;42: 203–219.
6. Vingarzan R, Belzer W, Thompson B. Nutrient levels in the atmosphere of the Elk Creek Watershed, Chilliwack, BC (1999-2000). Environment Canada technical report. 2002. Aquatic and atmospheric sciences division, Vancouver, Canada. Available from: http://www.pyr.ec.gc.ca/GeorgiaBasin/reports/NAECWC/GB-02–038_E.pdf
7. Johnson S, Fredriksen R. Precipitation and dry deposition chemistry concentrations and fluxes, Andrews Experimental Forest, 1969 to present. Long-Term Ecological Research. Forest Science Data Bank, Corvallis, OR. [Database] 2012 [cited 18 May 2014]. Available from: <http://andrewsforest.oregonstate.edu/data/abstract.cfm?dbcode=CP002>
8. Mahowald N, Jickells TD, Baker AR, Artaxo P, Benitez-Nelson CR, Bergametti G, et al. Global distribution of atmospheric phosphorus sources, concentrations and deposition rates, and anthropogenic impacts. Global Biogeochem Cycles. 2008 Dec 1;22(4).
9. Zhu JZ, Mazumder A. Estimating nitrogen exports in response to forest vegetation, age and soil types in two coastal-forested watersheds in British Columbia. Forest Ecol Manag. 2008;255(5-6): 1945–1959. doi:10.1016/j.foreco.2007.12.023
10. Schaefer SC, Hollibaugh JT, Alber M. Watershed nitrogen input and riverine export on the west coast of the US. Biogeochemistry. 2009;93(3): 219–233. doi:10.1007/s10533-009-9299-7
11. Sprague JB. Apparent sources of phosphorus affecting Cusheon Lake, Salt Spring Island, BC. A background report for the Cusheon Lake Watershed Management Plan and Steering Committee. Submitted by Sprague Associates Ltd: Salt Spring Island (BC). 2007. Available from: <http://www.islandstrust.bc.ca/media/341762/ssrptcusheonlkphosphorus.pdf>
12. Robinson M. Cultus Lake Aquatic Stewardship Strategy (CLASS) 5-year report. Fraser Basin Council. 2012. Available from: <http://cultusstewards.ca/wp-content/themes/Vizio-1.7.1/images/media/report-FSWP-2012.pdf>
13. National Audubon Society. The Christmas bird count historical results [Online]. 2010 [cited 13 Apr 2014]. Available from: <http://www.christmasbirdcount.org>
14. Environment Canada. North American Breeding Bird Survey-Canadian Trends Website, Data-version 2011. Gatineau (QC). 2013b. Available from: https://wildlife-species.canada.ca/breeding-bird-survey-results
15. Putt, AE. Spatiotemporal nutrient loading to Cultus Lake: Context for eutrophication and implications for integrated watershed-lake management. Report No. 591. School of Resource and Environmental Management, Faculty of Environment, Simon Fraser University. 2014. Available from <http://rem-main.rem.sfu.ca/theses/PuttAnnika_2014_MRM591.pdf>
16. Urban Systems Ltd. Cultus Lake South Sewerage Planning Study. Prepared for the Fraser Valley Regional District; USL File No. 0999.0042.01. Chilliwack (BC). 2012. Available from: <https://www.yumpu.com/en/document/view/41666790/cultus-lake-south-sewerage-planning-study-fraser-valley-/3>
17. ISL Engineering and Land Services. Cultus Lake sanitary sewer infrastructure assessment. Report submitted to the Fraser Valley Regional District, September 2012. Report No. 30825. 2012.
18. British Columbia Parks. 2011/12 Statistics Report. Victoria (BC). 2012.
19. Fraser Valley Regional District. Choices for our future: regional growth strategy for the Fraser Valley Regional District. Chilliwack (BC). 2004. Available from: <http://www.fvrd.ca/assets/Government/Documents/RGS%202004%20Choices%20for%20our%20Future.pdf>
20. Government of Canada. Migratory Birds Regulations, CRC, c 1035; 2014 [cited 2 Mar 2014]. Available from <http://canlii.ca/t/522gh>
21. Baxter AT, Robinson AP. A comparison of scavenging bird deterrence techniques at UK landfill sites. Int J Pest Manag. 2007;53(4): 347–56. doi:10.1080/09670870701421444
22. Cook A, Rushton S, Allan J, Baxter A. An evaluation of techniques to control problem bird species on landfill sites. Environ Manag. 2008;41(6): 834–843. doi:10.1007/s00267-008-9077-7
23. Thiériot E, Molina P, Giroux J-F. Rubber shots not as effective as selective culling in deterring gulls from landfill sites. Appl Anim Behav Sci. 2012;142: 109–115. doi:10.1016/j.applanim.2012.09.008
